# Short-Term Outcomes and Quality-of-Life Assessment Following Rives-Stoppa and Transversus Abdominis Release Procedures of Open Ventral Hernia Repair

**DOI:** 10.7759/cureus.41637

**Published:** 2023-07-10

**Authors:** Rajiv Kumar, Prem Prakash, Seema R Sinha, Nadeem Ahmad, Kanchan S Baitha

**Affiliations:** 1 General Surgery, Indira Gandhi Institute of Medical Sciences, Patna, IND; 2 Biochemistry, Indira Gandhi Institute of Medical Sciences, Patna, IND

**Keywords:** quality of life, recurrence, carolina comfort scale, rives-stoppa, tar, ventral hernia

## Abstract

Introduction: Ventral hernia is one of the common surgical conditions that can significantly impact a patient's quality of life (QoL). Open ventral hernia repair using the Rives-Stoppa (RS) and Transversus Abdominis Release (TAR) procedures has gained recognition for its effectiveness in achieving hernia repair and reducing the risk of further recurrence. However, limited research has been performed to explore the short-term outcomes and QoL assessment following these two surgical techniques. The aim of this study was to know the result after RS and TAR methods of hernia repair in terms of short-term recurrences, pain, postoperative complications, and QoL.

Methods: This was a prospective, interventional study, which included 30 patients fulfilling the inclusion criteria. The study group was subjected to posterior component separation (PCS)-TAR and RS repair as per surgical indication (RS if defect size 4-10cm; PCS-TAR if defect size >10cm and </= 15cm). All post-operative patients were followed up at postoperative day (POD) seven, POD 30, and POD 90 for postoperative pain, complications, and QoL using the hernia-specific Carolina Comfort Scale (CCS). At the same time, recurrence was studied till POD 180.

Results: Not a single recurrence was observed till POD 180 in either of the repair methods. The mean operative time for RS repair was 170.47 ± 15.08 minutes while for TAR repair was 188.8 ± 22.04 (p-value= 0.013). Surgical site infection (SSI) was reported in 14.28% of RS repair cases and 11.11% of TAR repair cases. Seroma formation was observed in 9.5% of RS repair cases. RS repair has less mean CCS score than TAR. The one-way ANOVA showed *f*-ratio=421.43 and p-value=0.00001 for RS repair while *f*-ratio= 298.05 and p-value=0 .00001 for TAR repair at POD seven, POD 30, and POD 90. Both RS and TAR repair markedly reduced mean scores in all three domains on POD 90.

Conclusion: Both RS and TAR had no recurrence in a short period of six months. The intraoperative time taken in TAR was less than in earlier studies. QoL improved postoperatively in both the repairs with RS repair having better QoL than TAR repair.

## Introduction

A ventral hernia is defined by a protrusion through the anterior abdominal wall fascia. Ventral abdominal wall hernias present a growing challenge that complicates 11-23% of all abdominal laparotomies. The ability to perform a reliable, durable ventral hernia repair with low morbidity and recurrence rate has become a significant problem for today’s general surgeon [1,2], as hernia repair failure rates range from 25% to 54% for primary suture repair, and up to 32% for open mesh repair [3,4]. The repair of ventral hernias has dramatically evolved over time. When mesh was introduced, the failure rate was seen to decrease from more than 60% to as low as 2% [5-7].

Rives-Stoppa (RS) repair evolved as an effective repair with favorable outcomes and low morbidity. The posterior rectus sheath dissection provides release of the rectus muscle and a well-vascularised “box” for mesh placement. This sublay mesh technique is increasingly becoming the world’s standard approach to the complex repair of ventral hernias, due to its durability and long-term outcomes in addition to the fact that mesh is excluded from the visceral contents and thus does not pose a problem for future abdominal surgery [8]. But the procedure is not appropriate for large defects due to its frequent inability of anterior fascial closure, which leads to large surfaces of mesh under the skin. As the number of large and complex abdominal wall defects is increasing, it is obvious that the procedure is not adequate for such pathology.

In 2012, Novitsky et al. reported a novel approach to posterior component separation by Transversus Abdominis Muscle Release (TAR) [9]. This is a lateral extension of RS repair with the creation of a wide space between the transversus abdominis (TA) muscle and fascia transversalis peritoneum complex. Complex ventral hernia repair is a frequent and challenging topic. Reconstructive techniques are numerous but most of them are unable to achieve the goals of hernioplasty. Posterior component separation (PCS) with TAR is a novel approach that offers a solution for complex ventral hernias [10].

In the last decade, the focus in hernia research has shifted from surgical outcomes such as recurrences and complications to patient-centered outcomes. Currently, chronic pain and quality of life (QoL) outcomes are frequently used as primary outcomes [11-13]. The concept of patient-reported outcomes serves to evaluate patients' points of view on outcomes [14]. The success of incisional hernia repair can be determined by adhering to the patient’s reported outcomes. Currently, there is no standardization of patient-reported outcomes in incisional hernia surgery, and methodological QoL instruments are poor [15].

Since large ventral hernias frequently occur in our region, there was a need to study this with respect to surgical outcomes of different procedures used in the repair of the large ventral hernia. Data regarding the advantages or disadvantages of different procedures are sparingly available; therefore, they need to be evaluated, and guidelines for efficient management need to be formulated. The present study was thus undertaken to assess the short-term surgical outcomes in terms of recurrence, pain, complications, and QoL after RS and TAR procedures in the repair of the ventral hernia.

## Materials and methods

Patient population

In this prospective interventional study, the population consisted of a total number of 30 patients reporting to the Department of General Surgery, Indira Gandhi Institute of Medical Sciences (IGIMS), Patna, a tertiary care hospital in Bihar, India. The study duration was two years from December 2020 to December 2022. This study was approved by the Institute Ethics Committee, IGIMS, Patna (approval memo number: 2004/IEC/IGIMS/2020, dated December 18, 2020). Written informed consent was also obtained from all the patients before enrolling them in the study after a clear explanation in their own language.

Study method

Data collection included detailed history, thorough clinical examination, contrast-enhanced computed tomography (CECT), and ultrasonography (USG) of the abdomen. The inclusion criteria consisted of both males and females aged 18-70 years falling under the American Society of Anesthesiologists (ASA) classification I and II with midline ventral hernias (primary and secondary) and defect size ranging from 4-15 cm. Patients who did not give consent or with comorbid conditions like malignant hypertension, chronic obstructive pulmonary disease (COPD), heart diseases, body mass index (BMI) >37.5 kg/m^2^, having active infections, sinus or fistula at hernia site, stoma, strangulated hernia, and pregnant or lactating were excluded from the study.

The patients presenting with ventral/incisional hernia under the study group were subjected to PCS-TAR using standard technique and RS repair as per surgical indication (RS if defect size 4-10cm and PCS-TAR if defect size >10cm and </= 15cm). The Follow-up period was the post-operative day (POD) seven, POD 30, and POD 90 for pain, complications, and QoL, while short-term recurrence was observed till POD 180. Recurrence was determined by clinical examination. USG was also done to access any local recurrence at POD 30 and POD 180. Acute postoperative pain by a visual numeric scale (VNS) was applied, which has been used in previous studies [16]. Post-operative complications namely hematoma, surgical site infection (SSI), seroma, sinus and fistula formation, and mesh explantation were studied till POD 90. Following discharge, patients were followed on POD seven by hospital revisit, and POD 30 and POD 90 through an email questionnaire or telephonic conversation (interview) as per the patient’s convenience to assess the Qol by the Carolina Comfort scale (CCS). The CCS score was derived by adding the scores from each of the 23 items (Table 1). The total score is based on a scale of 0-115; the best possible score is 0 and the worst possible score is 115.

**Table 1:** Carolina Comfort Scale 0 = No Symptoms, 1 = Mild but not bothersome symptoms, 2 = Mild and bothersome symptoms, 3 = Moderate and/or daily symptoms, 4 = Severe symptoms, 5 = Disabling symptoms, N/A = Activity was not performed

	English							
1.	While laying down, do you have							
a)	sensation of mesh	0	1	2	3	4	5	N/A
b)	pain	0	1	2	3	4	5	N/A
2.	While bending over, do you have							
a)	sensation of mesh	0	1	2	3	4	5	N/A
b)	pain	0	1	2	3	4	5	N/A
c)	movement limitations	0	1	2	3	4	5	N/A
3.	While sitting up, do you have							
a)	sensation of mesh	0	1	2	3	4	5	N/A
b)	pain	0	1	2	3	4	5	N/A
c)	movement limitations	0	1	2	3	4	5	N/A
4.	While performing activities of daily living (i.e. getting out of bed, bathing, getting dressed), do you have							
a)	sensation of mesh	0	1	2	3	4	5	N/A
b)	pain	0	1	2	3	4	5	N/A
c)	movement limitations	0	1	2	3	4	5	N/A
5.	When coughing or deep breathing, do you have							
a)	sensation of mesh	0	1	2	3	4	5	N/A
b)	pain	0	1	2	3	4	5	N/A
c)	movement limitations	0	1	2	3	4	5	N/A
6.	While walking, do you have							
a)	sensation of mesh	0	1	2	3	4	5	N/A
b)	pain	0	1	2	3	4	5	N/A
c)	movement limitations	0	1	2	3	4	5	N/A
7.	When walking up the stairs, do you have							
a)	sensation of mesh	0	1	2	3	4	5	N/A
b)	pain	0	1	2	3	4	5	N/A
c)	movement limitations	0	1	2	3	4	5	N/A
8.	While exercising, do you have							
a)	sensation of mesh	0	1	2	3	4	5	N/A
b)	pain	0	1	2	3	4	5	N/A
c)	movement limitations	0	1	2	3	4	5	N/A

Statistical analysis

Data was entered in the proforma, tabulated, and analyzed. The result was analyzed in terms of mean with standard deviation (SD), median, and proportions. Descriptive statistics were used to summarize the data as means with corresponding SD for continuous variables and percentages for categorical variables. Categorical variables were evaluated using Pearson’s chi-squared and exact tests where appropriate. A p-value of < 0.05 was considered statistically significant. All data were analyzed using IBM SPSS Statistics for Windows, Version 23.0 (Released 2015; IBM Corp., Armonk, New York, United States) as well as SAS version 9.4 (SAS Institute, Inc., Cary, North Carolina, United States).

## Results

A total of 30 patients with a mean age of 44.17 years and the median age of 45 years were included in the study. The sex distribution was 56.66% female and 43.33% male. The mean BMI of the included patients stood at 31.21 Kg/m^2^. A total of 21 (70%) patients were treated with RS repair and nine (30%) patients were treated with TAR repair. The demographic characteristics of the patients included in the study are depicted in Table 2. The mean transverse and longitudinal defect was 5.9±0.88 cm and 8.33 ±0.57 cm for RS repair. Whereas, TAR repair was performed for mean transverse and longitudinal defect sizes of 11.55 ±0.95 cm and 13.66 ±0.86 cm, respectively. The mean BMI of patients that underwent RS repair was 30.95 Kg/m^2^ while for TAR repair was 31.82 Kg/m^2^. Hypertension was reported in 38.1% and 33.3% of RS and TAR repair patients, respectively (P=0.8). Type 2 diabetes mellitus was observed in 21.05% of RS repair patients and 22.2% of TAR repair (P=0.9). Other comorbidities like hypothyroidism were observed in 4.7% and 3.33% of patients that underwent RS and TAR repair, respectively (P=0.03).

**Table 2:** Demographic characteristics of the patients included in the study RS: Rives-Stoppa; TAR: Transversus Abdominis Release

Variables	RS repair (N=21)	TAR repair (N=9)	p-value
Age, mean (SD)	42.57 (8.8)	47.88 (7.9)	0.13
Sex, n (%)			
Male	7 (33.3%)	5 (55.6%)	0.25
Female	14 (66.7%)	4 (44.4%)	
Obesity (BMI>30 Kg/m^2)^, n (%)			
Yes	13 (61.9%)	7 (77.7%)	0.39
No	8 (38.1%)	2 (22.3%)	
Smoking, n (%)			
Yes	3 (14.28%)	3 (33.3%)	0.23
No	18 (85.75%)	6 (66.7%)	
Hypertension, n (%)			
Yes	8 (38.1%)	3 (33.3%)	0.80
No	13 (61.9%)	6 (66.7%)	
Type 2 diabetes mellitus, n (%)			
Yes	5 (21.05%)	2 (22.2%)	0.90
No	16 (78.94%)	7 (77.8%)	
Other comorbidities			
Hypothyroidism, n (%)			
Yes	1 (4.7%)	3 (33.3%)	0.03
No	20 (95.3%)	6 (66.7%)	
Transverse diameter of defect, mean (SD)	5.9 (0.88)	11.55 (0.95)	0.0001
Longitudinal diameter of defect, mean (SD)	8.33 (0.57)	13.66 (0.86)	0.0001

**Figure 1:**
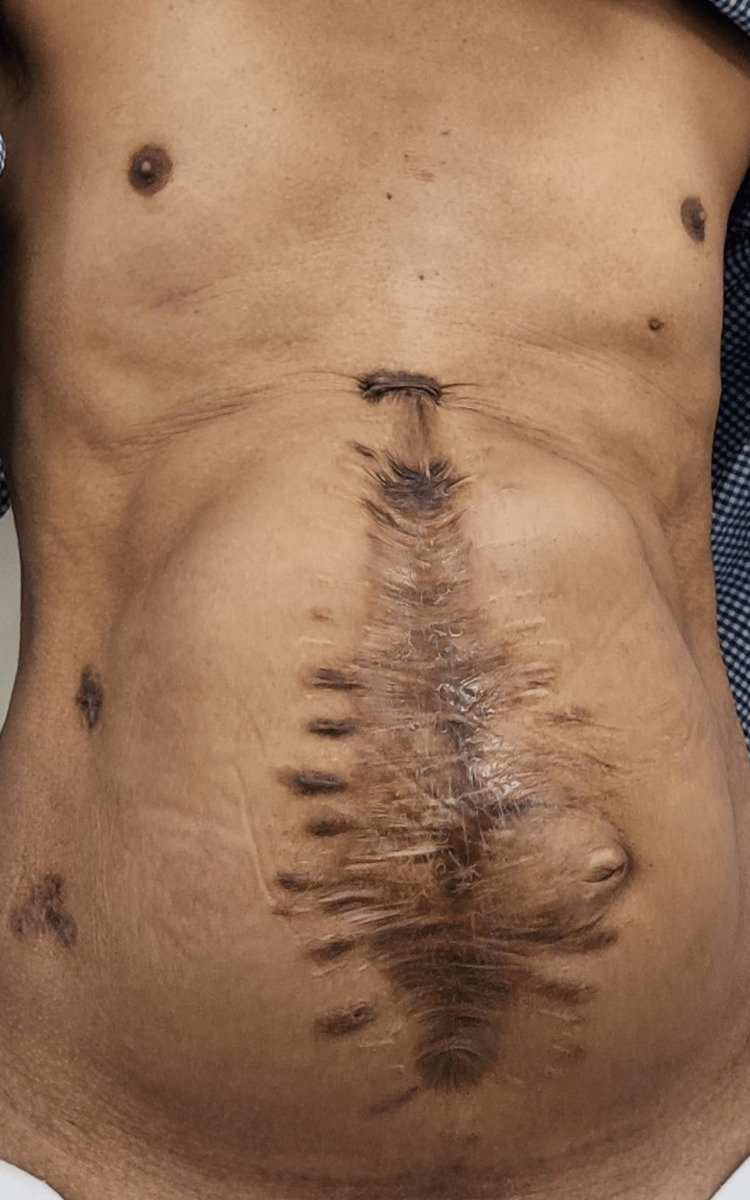
Preoperative image of a patient with large ventral hernia

**Figure 2:**
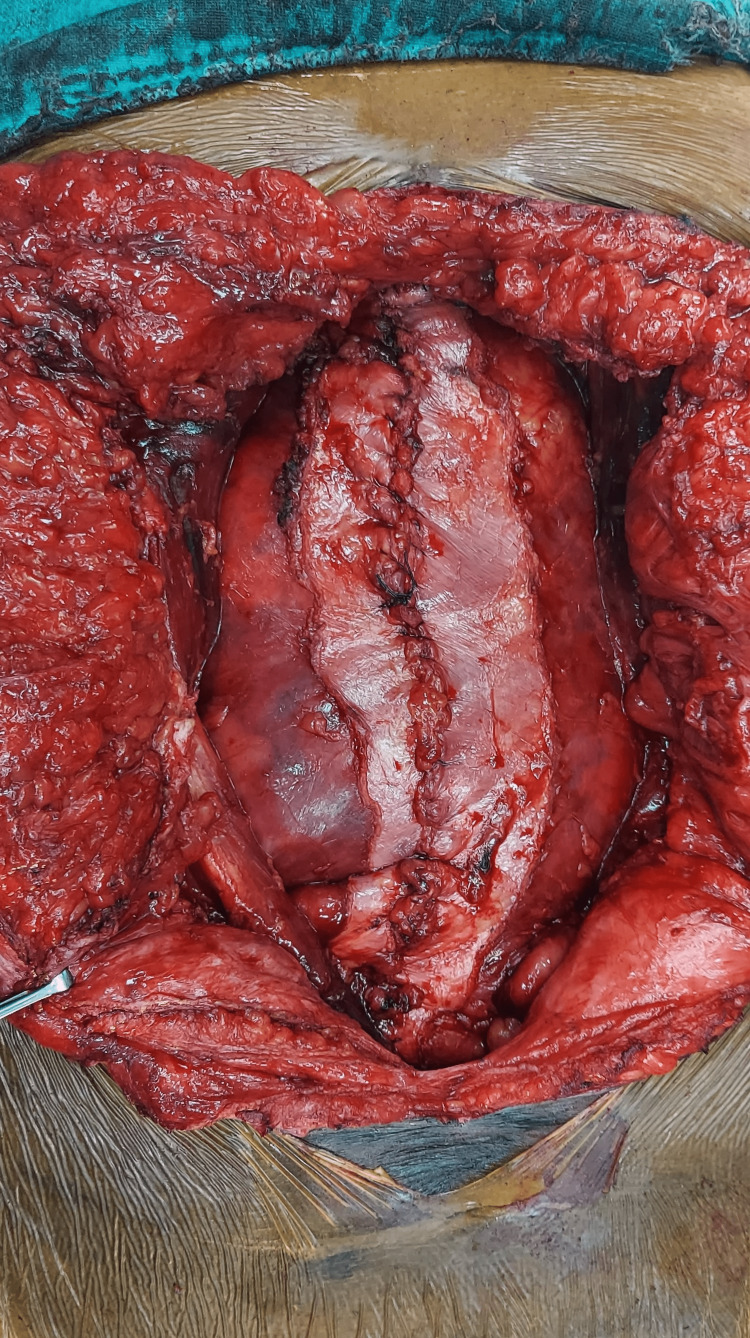
Intraoperative image of posterior component separation with transverses abdominis muscle release procedure

**Figure 3:**
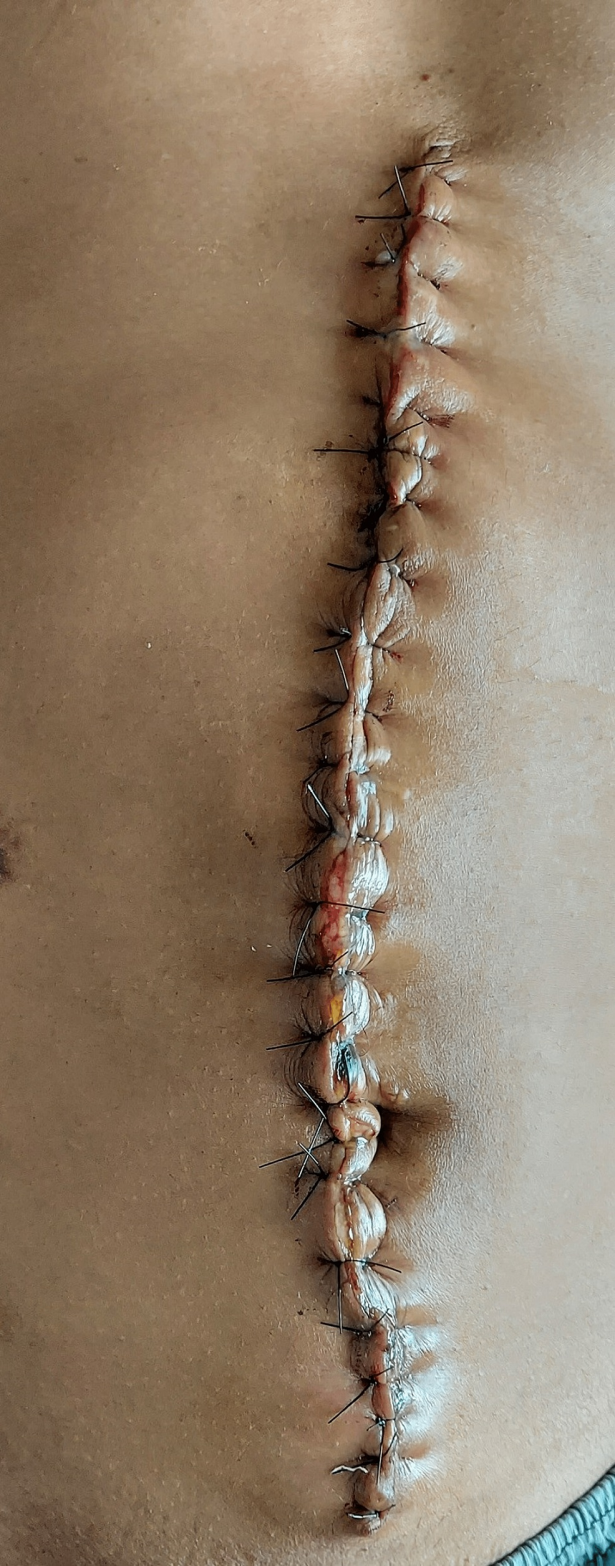
Post-operative image after the posterior component separation with transverses abdominis muscle release repair

The intraoperative variables for the patients that underwent hernia repair by either procedure are depicted in Table 3. The mean operative time for RS repair was 170.47 ± 15.08 minutes, which was less than the mean time of 188.8 ± 22.04 minutes for TAR repair (P=0.013). The mesh was placed in all the repairs using the sublay technique. The preoperative, intraoperative, and postoperative images of a patient with TAR repair for a large ventral incisional hernia are represented in Figures 1-3.

**Table 3:** Intraoperative and postoperative variables of the patients included in the study RS: Rives-Stoppa; TAR: Transverse Abdominis Release; POD: postoperative day; VNS: visual numeric scale *Ethicon, Inc., Raritan, New Jersey, United States **Meril Life Sciences Pvt. Ltd, Mumbai, India ***Lotus Surgicals Private Limited, Dehradun, Uttarakhand, India ****Becton, Dickinson and Company, Franklin Lakes, New Jersey, United States

Variables	RS repair (N=21)	TAR repair (N=09)	p-value
Intraoperative Variables			-
Mesh size (cm)			
15X15	10 (47.6%)	0	
15X20	10 (47.6%)	0	
25X25	1 (4.8%)	6 (66.7%)	
30x30	0	3 (33.3%)	
Average surgical time (minutes), mean (SD)	170.47 (15.08)	188.8 (22.04)	0.013
Mesh type			
Microporous	17 (80.9%)	7 (77.8%)	0.84
Macroporous	4 (19.1%)	2 (22.2%)	
Mesh Brand			
Ethicon*	10 (47.6%)	5 (55.6%)	-
Meril**	5 (23.8%)	2 (22.2%)	
Lotus***	4 (19%)	1 (11.1%)	
BARD****	2 (9.5%)	1 (11.1%)	
Swiss cheese formation	6 (30%)	4 (44.4%)	0.39
Divarication	5 (23.8%)	4 (44.4%)	0.26
Post-Operative Variables			
POD 1 pain score VNS scale, mean (SD)	7.16 (0.87)	7.55 (0.88)	0.27
Time to mesh drain removal in days, mean (SD)	2.6 (0.51)	2.6 (0.71)	1.0
Time to subcutaneous drain removal in days, mean (SD)	3.95 (0.60)	4 (0.50)	0.82
Hospital stay in days, mean (SD)	5.16 (0.58)	5.55 (0.41)	0.27
Surgical site infection	03 (14.28%)	01 (11.11%)	0.81
Seroma	2 (9.5%)	0	-
Haematoma	-	Not found	-
Flap necrosis	-	Not found	-

The mesh type commonly used was microporous (80%) and only 20% macroporous mesh was used. Only synthetic mesh was used in hernia repair by either of the techniques. The most commonly used brand of mesh was Ethicon (Ethicon, Inc., Raritan, New Jersey, United States) (50%) followed by Meril (Meril Life Sciences Pvt. Ltd, Mumbai, India) (23.33%). The mesh size varied depending on the defect size, and mostly the sizes of 15x15 cm^2^ (33.33%) and 15x20 cm^2^ (33.33%) were used. In the case of TAR repair, mesh sizes up to 30x30 cm^2^ (33.33%) were also used. Intraoperative European Hernia Society (EHS) classification-based defect sites were determined that included M1 (10%), M2 (6.6%), M3 (30%), M4 (20%), and M5 (16.67%) in the midline or medial region, while the lateral hernia included L1 (3.3%), L2 (10%), and L3 (3.3%). The presence of Swiss cheese formation was found in 44.44% and 30% of hernia cases were repaired with TAR. The Swiss cheese formation was not significant in the type of repair done (P= 0.84). Patients were followed up till 180 days post-operative. No hernia recurrence in any of the repairs was observed during the study period till POD 180.

The post-operative outcome was studied up to 90 days after surgery for complications and QoL. The mean time for mesh drain removal was the same in TAR repair and RS repair, i.e. 2.6 days. The mean hospital stay was 5.16 days and 5.55 days for RS and TAR repairs, respectively. No major complication such as mortality was observed in the study. SSI was reported in three (14.28%) cases of RS repair and one (11.11%) case of TAR repair. Haematoma formation was not reported in any of the cases. Seroma formation was observed in two (9.5%) cases of RS repair. Post-operative acute pain was scored using the VNS scale that ranged from 0 (best imaginable) to 10 (worst imaginable). The mean pain score on the VNS scale was 7.16 (0.87) for RS repair and 7.55 (0.88) for TAR repair. No sinus or fistula formation was observed in any of the repatriated cases. Mesh explantation was not done in any of the cases. The post-operative variables are depicted in Table 3.

The health-related short-term outcome of the QoL was studied using the CCS consisting of a specific questionnaire. The patients were asked to fill out the questionnaire on POD seven, POD 30, and POD 90. The completion rate was 100% on all three days for both RS and TAR repair. The mean score with standard deviation for RS and TAR repairs is given in Table 4.

**Table 4:** Mean CCS score for patients that underwent hernia repair CCS: Carolina Comfort Scale; POD: post-operative day; RS: Rives-Stoppa; TAR: Transversus Abdominis Release

Time point	RS, mean±SD	TAR, mean±SD	p-value
POD 07	62.52 ± 5.01	74.67 ± 4.24	0.0001
POD 30	45.28 ± 5.19	57.78 ± 5.38	0.0001
POD 90	23.8 ± 2.01	25.33 ± 3.1	0.116

## Discussion

The primary objective of the study was to assess the short-term recurrence after RS and TAR procedures in the repair of the ventral hernia up to six months (180 days) postoperatively. A total of 30 patients as per the inclusion criteria were studied out of these 21 patients underwent RS repair for defect size <10 cm and nine patients were treated with TAR repair (Defect size >10 cm and <=15 cm). The data was collected on multiple parameters, including the type of repair, size of the hernia, number of hernia defects (Swiss cheese type defects), divarication, type of mesh, size of the mesh, patient comorbidities, patient BMI, recurrence, and complications. The mean age of patients was 47.88 ±7.9 years for TAR repair while 42.57 ± 8.8 years for RS repair.

Not a single recurrence was observed in the study in either RS or TAR repair methods up to the period of 06 months (POD 180). This no recurrence can be attributed to previous studies that reported low recurrence rates within one year post-surgery [10, 17-18]. Similarly, Bueno et al. stated that recurrences occur after a mean period of 19.4 months [19]. The use of the component separation technique (CST) in hernia repair also leads to a low recurrence rate [20,21]. Another study stated that smoking, diabetes, COPD, ASA grade III-IV use, and steroids are predictors for the recurrence of hernia [22]. Most of these predictors were either excluded or not significant and therefore could have led to no recurrence during the period of study. Similarly, a study by Christophersen et al. also suggested that the hernia repair performed by high-volume surgeons seemed to have lower rates of hernia recurrence [23].

The intra-operative variables like the mean operative time for the repair of a large ventral hernia were 176 mins. The mean operative time for RS repair was 170.47± 15.08 mins while for TAR repair was 188.8 ± 22.04 mins. The time taken in TAR repair was less than the previous studies that reported 252 mins that depends on the surgical skills of the centre [24].

The mesh was laid by using the sublay technique and mostly microporous (80%) mesh was used. The microporous mesh was used due to cost-effectiveness, as most patients were from low economic backgrounds. The present study states that there was no recurrence but SSI was reported in three cases of RS repair and one case of TAR repair. Similar findings have been studied earlier that the non-absorbable synthetic microporous mesh implantation improved the observed hernia recurrence rates but is associated with increased wound complications in open hernia repair like surgical site infection. Park et al. observed that the lowest SSI rate belonged to non-smokers with BMI < 24.2 kg/m^2^ (1.9%), and the highest SSI to smokers with BMI > 42.3 kg/m^2^ (12%) [25]. The present study's mean BMI of patients was 31.21 Kg/m^2^ and smokers were also included hence it could have led to the SSI.

Seroma formation was observed in only Two (9.5%) cases of RS repair as it is sublay technique that leads to less seroma as studied by Beckers et al.whereas the onlay technique is more prone to a seroma [26].

The mean time to mesh drain removal in TAR repair and RS repair was the same ie: 2.6 days. The mean time for subcutaneous mesh removal was four days. The drains were removed only when the drainage stopped or became less than 25 ml. According to the study by Luo et al., there was no consensus on the duration the drain should stay in, with most surgeons averaging less than five days [27]. The length of hospital stay was similar to the previous study at 5.4 days in both repair techniques [28].

The QoL was assessed postoperatively up to POD 90 using a questionnaire-based CCS score. The complete response rate of patients was 100 % on POD 07/POD 30/POD 90 for both the repairs as it is well accepted by patients the response and completion rate was high. The completion rate did not correlate with the type of hernia repair. The present study showed the *P *value of 0.0001 in RS and TAR repair postoperative QoL on POD 07 and POD 30. Whereas this was statistically different on POD 90 (P= 0.116). After hernia repair, there was no statistically significant difference in pain and movement score in RS and TAR repair techniques. RS group had a higher mesh sensation score at POD 07. Rives-Stoppa technique with sublay mesh position where lack of innervated tissue may have led to increased mesh sensation [29]. The highest incidence of pain was in exercising followed by bending over. A one-way ANOVA was performed to compare the effect of RS repair and TAR repair on the CCS score on POD 07, POD 30, and POD 90. The study of Balla et al. stated that the trend of decreasing postoperative pain and activity limitation was significant over time for all CST methods which was significant for both RS and TAR repair in the present study with P= 0.00001 at POD seven and POD 30 [30]. The mean CCS score for RS repair at POD 07/POD 30/POD 90 was less than the TAR CCS score, this is because of more mesh sensation, pain, and movement limitation in TAR repair. Moreover, RS and TAR CCS were statistically significant at POD 07 and POD 30 (P= 0.0001) but not at POD 90 (P= 0.116).

Limitation

However, the limitation of this study was that it was conducted at a single center, which may limit the generalizability of the findings to a broader population. The patient demographics, surgical techniques, and postoperative care protocols at the specific center may not be representative of other institutions, potentially affecting the external validity of the results. The study may have had a limited sample size, which could affect the statistical power and precision of the results. 

## Conclusions

No recurrence was observed in the short duration of 180 days post-operatively in both RS and TAR repair techniques. The postoperative outcomes in terms of pain, complications like SSI, and seroma formation were also less in both methods. The duration of TAR repair was less as compared to earlier studies. QoL improved in both the methods but RS repair showed less CCS score indicative of better quality of life as compared to TAR repair.
